# Case report: Severe brain hypoxic damages after acute methanol poisoning

**DOI:** 10.1002/ccr3.5715

**Published:** 2022-04-12

**Authors:** Ameni Abidi, Asma Souid, Khalaf B Abdallah, Nadia Hammami, Selima Siala, Nozha Brahmi, Mohamed B Hamouda

**Affiliations:** ^1^ Department of Intensive care and Toxicology Centre d'assistance médicale d'urgence CAMU Tunis Tunisia; ^2^ Department of Neuroradiology Mongi Ben Hamida National Institute of Neurology Tunis Tunisia

**Keywords:** case report, hemorrhagic necrosis, hypoxia, magnetic resonance imaging, methanol poisoning, white matter demyelination

## Abstract

Methanol poisoning is a challenging clinical situation with irreversible neurologic complication mainly encountered in developed countries. We report a case of a 50‐year‐old patient who presented with methanol poisoning, symptomatic of respiratory and neurologic failure. In this context, cerebral magnetic resonance imaging concluded entangled injury mechanisms leading to neurologic failure.

## Abstract 2

In methanol poisoning, cerebral magnetic resonance imaging should be performed to identify neurologic injury mechanisms and to adapt the management of the condition. Secondary hypoxia in acute methanol poisoning can worsen the final outcome.

## 1 INTRODUCTION

Hypoxic–ischemic brain injury is common after cardiac arrest. Severe brain damage can be observed after prolonged brain hypoxia following some intoxications, with certain specificities for methanol.^1^ Imaging and mainly magnetic resonance imaging (MRI) is done to assess the amount of central nervous system damage, the prognosis as well as the outcome. We present a case of severe putaminal hemorrhagic necrosis and diffuse white matter damage due to both methanol poisoning and severe hypoxia with a poor prognosis.

Through our case, we aim to demonstrate the role of cerebral MRI in detecting the different mechanisms of neurologic failure in order to adapt the management of this intoxication.

## 2 CASE REPORT

We present a case of a 50‐year‐old male patient, with a 20‐year history of addiction to alcohol and recent ingestion of an amount of cologne water instead of alcohol for economic reasons. He presented in our Tunis Centre d'assistance médicale d'urgence's emergency department, in April 2020, with blurred vision and diplopia 21 h after the ingestion of one liter of unusual, commercialized liquor containing 70% formalin. A few hours later, he lapsed into a coma and had respiratory distress with SpO_2_ of 77%. Mechanical ventilation was required for advanced airway management, and he was transferred into our ICU. Investigations showed severe metabolic acidosis: pH = 6.87, PaCO_2_ = 25 mmHg, HCO_3_‐ = 4.6 mmol/L and elevated anion gap of 27 mEq/L with hyperlactatemia of 5 mmol/L. Toxicology and drug screen in blood samples revealed high methanol levels of 6.23 g/L. The patient was given 40% ethanol as a bolus of 0.6 g/kg and a maintenance dose of 100 mg/kg/h in association with 42‰ bicarbonate perfusion, two hemodialysis sessions of 6 h each, blood pump speed of 200 ml/min, adjuvant treatment with intravenous folic acid 200 mg daily and intravenous Vitamin B1 300 mg daily.

**FIGURE 1:**
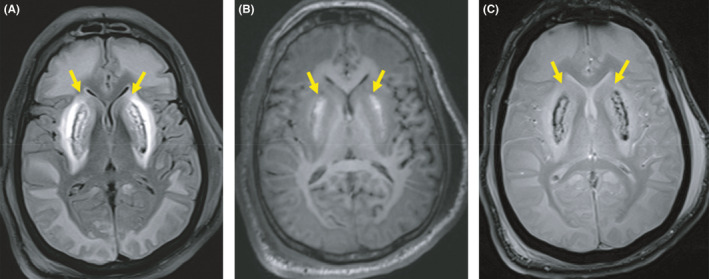
Axial brain magnetic resonance images showing putaminal hemorrhagic necrosis (arrows). (A) FLAIR sequence showing bilateral heterogeneous putaminal hyperintensities surrounded by hypointense borders. (B) T1‐weighted image: high signal on putaminal affected area representing the hemorrhagic nature. (C) Putaminal low signal regions on the T2* sequence related to a hemosiderin component. We note also the diffuse subcortical white matter demyelination (A–C)

**FIGURE 2:**
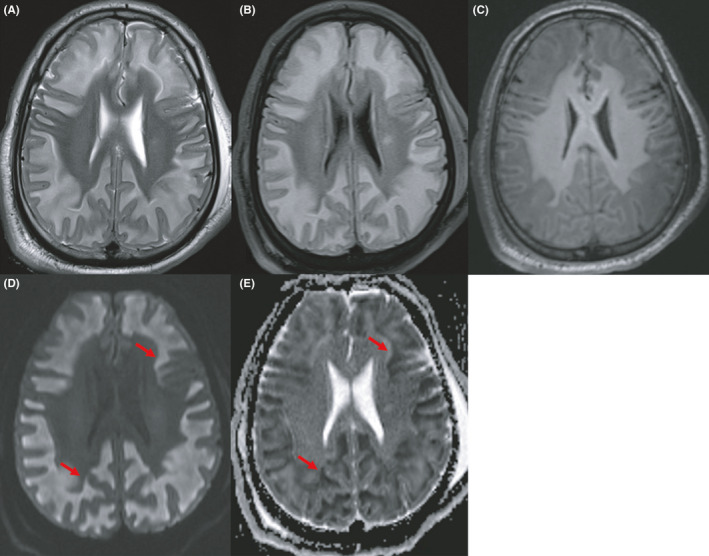
Axial brain magnetic resonance images showing diffuse subcortical white matter demyelination: (A + B) T2‐weighted image (A) and FLAIR sequence (B) showing bilateral diffuse subcortical white matter high signal, sparing subcortical U‐fibers and deep periventricular white matter. (C) Hypointense subcortical white matter on T1‐weighted image. (D + E) This extensive demyelination shows high signal intensity on B1000 diffusion‐weighted image (D) with low apparent diffusion coefficient (ADC) values (arrows)

Brain MRI performed on the seventh day showed bilateral symmetrical putaminal signal anomalies (Figure 1) consisting of heterogeneous hyperintensities in T1, T2, and fluid‐attenuated inversion recovery (FLAIR) sequences containing low signal regions on T2*‐weighted gradient echo and low apparent diffusion coefficient (ADC) value regions on diffusion‐weighted images. These findings represent the putaminal hemorrhagic necrosis. MRI showed also diffuse bilateral symmetrical extensive white matter abnormal signal intensity showing a T2/FLAIR hyperintensity, a T1 hypointensity with low ADC values on diffusion‐weighted images and respecting subcortical U‐fibers (Figure 2). There was no hemorrhagic stigma in the white matter. There was no restricted diffusion in the retrobulbar segment of the optic nerves. Written informed consent for publication of their clinical details and clinical images was obtained from the patient's family.

The electroencephalogram done on day 26 showed a slow disorganized, non‐reactive pattern, indicating diffuse brain suffering. The patient did not demonstrate neurological recovery. On day 32, refractory hypoxia under mechanical ventilation led to his death.

## 3 DISCUSSION

Putaminal hemorrhagic necrosis can represent an early consequence of cerebral hypoxia in general, but it is most often seen after carbon monoxide intoxication. However, this finding can also happen in isolated methanol intoxication.^1^ In our case, we cannot differentiate whether putaminal lesions were due to hypoxemia or specific methanol toxicity. In fact, formic acid formation secondary to methanol poisoning can inhibit cytochrome oxidase activity in the mitochondria, leading to histotoxic hypoxia with predilection for the brain and the visual pathway.^2^, ^3^ Reports have described an association between extensive white matter demyelination and basal ganglionic damage after hypoxia.^4^ This damage is explained in our case probably by the delay of resuscitation and, therefore, brain hypoxia. In fact, hypoxia causes necrosis and edema in different regions in the brain. Cerebral cortex, hippocampus, cerebral cortex as well as caudate nucleus, putamen, and globus pallidus are considered as vulnerable zones in the brain.^5^ Putamen is prone to develop injuries because of its high metabolic demand and its crucial place in vascular perfusion's boundary zones.^6^

The extension of these lesions depends on hemodynamic and metabolic patient characteristics but mostly the duration of hypoxia. Individuals older than 30 years like our patient are at greater risk of delayed demyelination.^7^

Evolution varies from recovery to death. In our case, the patient did not demonstrate neurological recovery.

As for other methanol intoxication MRI findings, optic nerve damage can be assessed by enhanced T1 fat‐saturated (FatSat) images and diffusion‐weighted images showing a bilateral mild contrast enhancement and restricted diffusion in the retrobulbar segment of the optic nerves.^8^ In our case, there was no restricted diffusion in the optic nerves.

Our case is interesting because it represents severe methanol intoxication associated with a severe hypoxic–ischemic brain injury. MRI was crucial for the diagnosis of this damage.

We attest the lack of a 2nd control MRI after antidote therapy, which could have given us a thorough understanding of the injury evolution in our patient.

## 4 CONCLUSION

In severe methanol intoxication, both brain prolonged hypoxia and the intoxication itself can lead to severe putaminal hemorrhagic necrosis. Diffuse white matter demyelination is secondary to the delay of resuscitation and, therefore, brain hypoxia. Magnetic resonance imaging is essential in identifying the entangled neurologic injury lesions and informing about the patient's prognosis.

## CONFLICT OF INTEREST

No competing interest were disclosed.

## AUTHOR CONTRIBUTIONS

All authors were involved in the researching, writing, and editing of the manuscript.

## CONSENT

Written informed consent for publication of their clinical details and clinical images was obtained from the patient's family.

## Data Availability

All data underlying the results are available as part of the article, and no additional source data are required.
